# Assessment of Health Indicators in Individuals with Intestinal Stoma using the Nursing Outcomes Classification: A Cross-Sectional Study

**DOI:** 10.3389/fsurg.2022.870379

**Published:** 2022-05-20

**Authors:** Noelia Moya-Muñoz, Elena Armenteros-Fernández, Clara Bautista-Mártir, Irene Del Pilar Vílchez-Díaz, Isabel María López-Medina, Rafael Montoya-Juárez, César Hueso-Montoro, Concepción Capilla-Díaz

**Affiliations:** ^1^ Virgen de las Nieves University Hospital, Granada, Spain; ^2^ Son Espasses University Hospital, Palma de Mallorca, Spain; ^3^ Virgen de la Luz Hospital, Cuenca, Spain; ^4^ Toledo University Hospital, Toledo, Spain; ^5^ Department of Nursing, Faculty of Health Sciences, University of Jaen, Jaen, Spain; ^6^ Department of Nursing, Faculty of Health Sciences, University of Granada; Instituto de Investigación Biosanitaria ibs.GRANADA, Granada, Spain

**Keywords:** colostomy, ileostomy, ostomy, standardized nursing terminology, nursing evaluation research

## Abstract

**Aim:**

To determine nursing outcomes in individuals with intestinal stoma and the relationships between them and sociodemographic and clinical variables.

**Design:**

Cross-sectional study performed with 102 subjects at the General Surgery Unit of a first-level hospital.

**Methods:**

Data on the presence of nursing outcomes were collected using the Nursing Outcomes Classification. Data on sociodemographic and clinical variables were also collected. Univariate and bivariate data analyses were performed.

**Results:**

Outcomes related to participation in making health decisions and knowledge of ostomy care were assessed across the study sample. Period of care (post-operative and follow-up) was the most common significant variable (*p* < 0.05) among the outcomes. The outcome scores ranged from 2 to 3, indicating a moderate level of impairment in the physical, psychological, and social spheres of these patients. The scores in the indicators on Participation in making health decisions and Knowledge of stoma care improved in the period of continuity of care compared to the postoperative period, being this difference statistically significant (*p* < 0.001).

**Conclusions:**

The care plan for individuals with intestinal stoma needs to include indicators measuring patient participation in making decisions related to their condition, as well as indicators related to their knowledge and self-care of their stoma. Relevance to clinical practice: This study aims to determine the nursing outcomes in individuals with intestinal stoma and the relationships between them and sociodemographic and clinical variables. It provides the opportunity to plan achievable objectives with patients using a system of indicators that facilitate their assessment and monitoring.

## Introduction

Colorectal cancer (CRC) is one of the most common diseases in Western countries. It is estimated that, in EU countries in 2020, colorectal cancer accounted for 12.7% of all new cancer diagnoses and 12.4% of all deaths due to cancer. Approximately 191,053 and 150,366 cases of colorectal cancer were diagnosed in Europe in men and women respectively, and around 87,185 men and 68,920 women died from this disease (1). In Spain, colon cancer was the second largest cause of cancer mortality in men and the third largest in women (2) with an estimate of 44,231 new cases for both sexes (3).

Treatments often result in the formation of an ostomy. The resection of a stoma is also performed in other conditions such as Inflammatory Bowel Disease (IBD), including ulcerative colitis or Crohn’s Disease, in abdominal trauma and in inherited disorders such as familial adenomatous polyposis. In Spain there are approximately 70,000 individuals with an ostomy and each year there are 16,000 new cases (4).

Nursing care plans are vehicles for communication. They are records of the care provided and are essential tools for day-to-day care. Care plans are structured according to the nursing care system in place and can therefore take various forms (5). Nursing care plans are essential for intestinal stoma patients as they need to adapt to their new situation. They need continuous care from the time they receive the news of the surgical procedure through the postoperative period and into the period of continuity of care (6).

Patients with intestinal stoma face a number of physical changes that are related to altered defecation patterns: altered body image and altered sexual function. They are also forced to make changes in diet, physical activity, leisure, work, and clothing. As well as having their physical aspects affected, patients often experience psychosocial problems, including depression, feelings of shame, anxiety, low self-esteem, isolation, and social stigma, which also affect their family environment (7).

Family support plays an important role in the acceptance of the stoma. The ability to actively listen to and support family and friends in dealing with the patient’s feelings helps them to overcome problems and to strengthen the family relationship. The attitude of the spouse in this situation is particularly important. Studies show that acceptance of body image improves sexual desire when partners are understanding and supportive. These patients usually experience a decrease in libido and sexual relations, which is related to lack of confidence and coadjuvant psychological alterations (8).

Self-care plays an important role in the patient’s own physical and psychosocial adaptation. Most individuals with an intestinal stoma have difficulties in self-care. The main problems patients encounter are stoma leaks, peristomal skin problems, and difficulty in stoma care, as they need to frequently change the pouching system, which takes some patients about 30 min (7, 9).

In order to provide patients with adequate nursing care, it is necessary to develop care plans that facilitate the standardisation of care while serving as a mechanism for communication between the different professionals involved. This must take place within the framework of the nursing process and taking into consideration the importance of care plan individualisation, thus ensuring the good quality of care by using a scientific method.

The nursing process incorporates standardised languages to reduce variability of care in clinical practice, which is how high quality and continuity of care are achieved. Currently, the most widely used nursing standardised languages are NANDA, which identifies the needs or problems in patient care; the Nursing Interventions Classification (NIC), which lists the interventions needed to address these problems; and the Nursing Outcomes Classification (NOC), which assesses the outcomes of these interventions (10).

The availability of standardised languages provides nurses with benefits related to improved communication, assistance in the delivery of patient care, increased visibility of the nursing profession through nursing interventions, nursing practice outcomes, and education of future nurses. The Nursing Outcomes Classification (NOC) is a standardised nursing language that has been translated into nine languages and has been used to explore and describe nursing practice in a variety of nursing practice specialisms, patient groups, and healthcare settings (11, 12). Recent works highlight that stoma therapy nurse demand the use of unified standardized indicators that provide sufficient data to measure, quantify and assess the level and quality of care provided (4).

## Methods

### Aim

To determine nursing outcomes (NOC) and their indicators in individuals with an intestinal stoma and the relationships between them and sociodemographic and clinical variables.

### Design

This study used a cross-sectional descriptive design, following the STROBE guidelines (13). It was conducted at the General Surgery Unit of the San Cecilio University Hospital in Granada, Spain. This unit includes post-operative and follow-up areas and, as such, provides care to patients from surgery to discharge. This unit also includes 82 inpatient beds and 5 operating theatres (two of them for surgical emergencies). Patients undergoing scheduled or urgent ostomy surgery are cared for in this hospital, which also includes a post-discharge follow-up unit.

### Sample

The study population included patients with an intestinal stoma who were admitted for surgery or receiving ongoing care. The exclusion criteria were as follows: patients under 16 years of age (in whom stomas are rare), patients with cognitive impairments that might hinder their assessment, and patients who did not wish to participate. Consecutive sampling was used until 102 subjects were recruited. Data were collected between February and April 2017. All subjects who agreed to participate in the study during that period were selected.

### Data Collection

Data on the following sociodemographic and clinical variables were collected: age (years), sex (male/female), membership in a patient association (yes/no), family member with an ostomy (yes/no), stoma site marking (yes/no), medical diagnosis (oncological/non-oncological), time with the stoma (less than one year/more than one year), and period of care (post-operative period and follow-up period). The post-operative period of care began when the patient was discharged from the recovery room and ended when the patient was discharged from the hospital. Then, the follow-up period began.

A series of 30 NOC outcomes and their indicators related to physical and psychological aspects of care were selected as main variables. The NOC was used to collect data on nursing outcomes. The presence or absence of NOC outcomes and their indicators was recorded based on a previously prepared list of outcomes. The process of selecting these outcomes and the list itself were based on a previous qualitative metasynthesis on the experiences of patients with intestinal stoma (14). Subsequently, the list was reviewed by three experts in nursing methodology and a stoma therapy nurse.

Each patient was assessed in the unit itself. To this end, a notebook was prepared with instructions, ethical considerations, the list of variables, and the outcomes selected for analysis. The data were collected in a brief interview conducted by a nurse with experience in stoma therapy and NOC terminology. During data collection, the nurse introduced themselves to the patient, explained the purpose of the study to them, requested their participation in the study, and handed them the informed consent form to sign. Once the patient had been assessed by the nurse, the nursing supervisor of the gastroenterology ward, an expert in stoma therapy, examined and verified the selected data. No patients refused to participate.

More details on the sample and data collection process can be found in two previous publications related to this research (15, 16).

### Data Analysis

Each NOC outcome includes a label, a definition, and a 4-digit code, as well as a list of indicators that are selected by nurses to assess the overall outcome of the care plan for each patient. These indicators are measured using a Likert scale, in which the intensity or frequency of a described situation is represented by a number from 1 to 5, where 1 is the worst possible situation for the patient regarding a given indicator and 5 is the best possible situation. Each indicator consists of 6 digits, the first four of which correspond to the digits of the NOC outcome to which they belong (12).

Data were collected in a Microsoft Excel spreadsheet and then exported to IBM Statistical Package for the Social Sciences (SPSS), version 23, for data analysis.

Firstly, the NOC outcomes and their indicators were analysed as binary variables (presence or absence thereof in each case) and expressed as absolute frequencies and percentages. Contingency tables and the chi-squared test were used to contrast the other variables with each NOC outcome and their indicators. Fisher’s exact test was used when the conditions required for the previous test were not met. Odds ratios and confidence intervals (95%) were taken as measures of effect size for statistically significant associations. Student’s t-test was used to compare each NOC outcome in relation to age, as age was normally distributed.

Secondly, the NOC indicator scores were analysed as continuous variables expressed as mean, standard deviation, median, IQR, and CI (95%). In addition, a bivariate analysis was performed using a non-parametric test because the distribution of the variables did not fulfil the normality criterion. Each NOC indicator was compared with the rest of the factors using the non-parametric Mann-Whitney U test. Age (as the only continuous factor) was compared with the NOC indicators using Spearman’s non-parametric correlation. This analysis was performed on the NOC outcomes which had 20 or more valid cases (recommended criterion in inferential analyses).

The normality of the continuous data was tested using the Kolmogorov-Smirnov test when the n of each subgroup was at least 50, or the Shapiro-Wilk test when the n of each subgroup was lower than 50.

The statistical significance threshold for all tests was set at *p* < 0.05.

### Ethical Considerations

This study was approved by the Research Ethics Committee of the province of Granada, Spain, under file number PI-0564-2011. Each patient was given a fact sheet with the research objectives. All patients gave their informed consent prior to their participation. Their data were handled with strict confidentiality at all times, ensuring patient anonymity by using an identification code. The nurse in charge of collecting the data ensured the confidentiality of the information.

### Validity and Rigour

The process of selecting these outcomes and the list itself were based on a previous qualitative metasynthesis on the experiences of patients with intestinal stoma. The responses and clinical judgement of the nurses participating in the study were used to determine the presence or absence of each outcome and to determine the score of the indicators in each case. Once the patient had been assessed by the head nurse, the gastroenterology ward nurse supervisor, an expert in stoma therapy, reviewed and verified the collected data. The decisions of which outcomes to include were then made on the basis of a judicious discussion between the head nurse and the nursing supervisor.

## Results

The sociodemographic and clinical characteristics of the sample are shown in Table 1.

**Table 1:** Sociodemographic and clinical characteristics of the sample.

Quantitative Variable	R	M	SD	Me	IQR
Age	33–84	62.57	11.3	64.50	55–71
Qualitative Variable	Frequency			%	
Sex					
Male	52			51.00%	
Female	50			49.00%	
Member of a patient association					
Yes	7			6.90%	
No	95			93.10%	
Family member with an ostomy					
Yes	16			15.70%	
No	86			84.30%	
Medical diagnosis					
Oncological	82			80.40%	
Non-oncological	20			19.60%	
Time with stoma					
The previous year	90			88.20%	
More than a year ago	12			11.80%	
Period of care					
Postoperative period	45			44.10%	
Follow-up period	57			55.90%	
Stoma site marking					
Yes	84			82.40%	
No	18			17.60%	

*R, Range; M, Mean; SD, Standard Deviation; Me, Median; IQR, Interquartile Range.*

### Distribution of the NOC Outcomes Identified and their Indicators

To assess the prevalence of each NOC outcome in the sample, the percentage of subjects presenting with that outcome was analysed (Table 2).

**Table 2:** Distribution of NOC outcomes and their indicators.

NOC code and indicators/label	Yes		No	
	*N*	%	*N*	%
0004 Sleep				
000404 Sleep quality	72	70.60%	30	29.4%
000421 Difficulty getting to sleep	72	70.60%	30	29.4%
0006 Psychomotor Energy				
000608 Exhibits stable energy level	29	28.40%	73	71.6%
000609 Exhibits ability to accomplish daily tasks	29	28.40%	73	71.6%
0119 Sexual functioning				
011901 Attains sexual arousal	22	21.60%	80	78.4%
011904 Uses assistive device as needed	0	0.00%	102	100%
011907 Expresses ability to perform sexually despite physical imperfection	23	22.50%	79	77.5%
011908 Expresses comfort with sexual expression	23	22.50%	79	77.5%
011909 Expresses self-esteem	21	20.60%	81	79.4%
011910 Expresses comfort with body	3	2.90%	99	97.1%
011911 Expresses sexual interest	19	18.60%	83	81.4%
011912 Expresses ability to be intimate	18	17.60%	84	82.4%
011913 Express willingness to be sexual	21	20.60%	81	79.4%
011921 Communicates comfortably with partner	21	20.60%	81	79.4%
0501 Bowel Elimination				
050101 Elimination pattern	3	2.90%	99	97.1%
1004 Nutritional Status				
100401 Nutrients intake	0	0.00%	102	100%
100403 Energy	22	21.60%	80	78.4%
100408 Fluid intake	22	21.60%	80	78.4%
100411 Hydration	22	21.60%	80	78.4%
1015 Gastrointestinal Function				
101501 Food tolerance	3	2.90%	99	97.1%
101503 Frequency of stools	8	7.80%	94	92.2%
101524 Appetite	8	78.00%	94	92.2%
1101 Tissue integrity: Skin and Mucous Membranes				
110102 Sensation	0	0.00%	102	100%
110108 Texture	0	0.00%	102	100%
110113 Skin integrity	66	64.70%	36	35.3%
110115 Skin Lesions	66	64.70%	36	35.3%
110121 Erythema	66	64.70%	36	35.3%
1200 Body Image				
120005 Satisfaction with body appearance	50	49.00%	52	51.0%
120014 Adjustment to body changes due to injury	50	49.00%	52	51.0%
1201 Hope				
120102 Expresses faith	0	0.00%	102	100%
120112 Sets goals	0	0.00%	102	100%
1205 Self-Esteem				
120501 Verbalizations of self-acceptance	33	32.40%	69	67.6%
120502 Acceptance of self-limitations	33	32.40%	69	67.6%
1212 Stress level				
121220 Irritability	0	0.00%	102	100%
121222 Anxiety	0	0.00%	102	100%
1300 Acceptance: Health status				
130002 Relinquishes previous concept of personal health	5	4.90%	97	95.1%
130010 Copes with health situation	5	4.90%	97	95.1%
1302 Coping				
130201 Identifies effective coping patterns	0	0.00%	102	100%
130210 Uses behaviors to reduce stress	31	30.40%	71	69.6%
130212 Use effective coping strategies	28	27.50%	74	72.5%
130213 Avoids unduly stressful situations	31	30.40%	71	69.6%
1304 Grief Resolution				
130411 Reports decreased preocupation with loss	2	2.00%	100	98.0%
130414 Reports adequate sleep	2	2.00%	100	98.0%
1308 Adaptation to Physical Disability				
130820 Reports increase in psychological comfort	0	0.00%	102	100%
1309 Personal Resiliency				
130906 Exhibits positive mood	34	33.30%	68	66.7%
130915 Proposes practical, constructive solutions for disputes	34	33.00%	68	66.7%
1402 Anxiety Self-Control				
140202 Eliminates precursor of anxiety	22	21.60%	80	78.4%
140205 Plans coping strategies for stressful situations	0	0.00%	102	100%
140206 Uses effective coping strategies	0	0.00%	102	100%
140207 Uses relaxation techniques to reduce anxiety	22	21.60%	80	78.4%
140210 Maintains role performance	20	19.60%	82	80.4%
140211 Maintains social relationships	22	21.60%	80	78.4%
140214 Maintains adequate sleep	22	21.60%	80	78.4%
140215 Monitors physical manifestations of anxiety	0	0.00%	102	100%
140217 Controls anxiety response	0	0.00%	102	100.00%
1404 Fear Self-Control				
140401 Monitors intensity of fear	4	3.90%	98	96.10%
140402 Eliminates precursors of fear	4	3.90%	98	96.10%
140404 Avoids source of fear when possible	0	0.00%	102	100.00%
140405 Plans coping strategies for fearful situations	3	2.90%	99	97.10%
140406 Uses effective coping strategies	4	3.90%	98	96.10%
140407 Uses relaxation techniques to reduce fear	4	3.90%	98	96.10%
140408 Monitors duration of episodes	0	0.00%	102	100.00%
140410 Maintains role performance	1	1.00%	101	99.00%
140411 Maintains social relationships	1	1.00%	101	99.00%
140414 Maintains physical fuctioning	0	0.00%	102	100.00%
140417 Control fear response	0	0.00%	102	100.00%
1503 Social Involvement				
150303 Interacts with family members	21	20.60%	81	79.40%
150311 Participates in leisure activities with others	21	20.60%	81	79.40%
1504 Social Support				
150405 Emotional assistance provided by others	0	0.00%	102	100.00%
150408 Willingnes to call on others for assistance	0	0.00%	102	100.00%
1606 Participation in Health Care Decisions				
160601 Claims decision-making responsibility	0	0.00%	102	100.00%
160603 Seeks reputable information	102	100.00%	0	0.00%
160604 Defines available options	102	100.00%	0	0.00%
160607 Identifies barriers to desired outcome achievement	0	0.00%	102	100.00%
160610 Identifies available support for achieve desired outcomes	102	100.00%	0	0.00%
1615 Ostomy Self-care				
161519 Expresses acceptance of ostomy	11	10.80%	91	89.20%
161523 Obtains assistance from a health professional	12	11.80%	90	88.20%
1701 Health Beliefs: Perceived Ability to perform				
170102 Perception that health behavior requires reasonable effort	8	7.80%	94	92.20%
170104 Perception of likelihood of performing health behavior is not excessive	4	3.90%	98	96.10%
170107 Confidence related to observation of successful experiences of others	8	7.80%	94	92.20%
170108 Confidence in ability to perform health behavior	8	7.80%	94	92.20%
1804 Knowledge: Energy Conservation				
180403 Appropriate activities	0	0.00%	102	100.00%
180407 Strategies to balance activity and rest	0	0.00%	102	100.00%
1829 Knowledge: Ostomy Care				
182901 Function of ostomy	101	99.00%	1	1.00%
182903 Skin care needs around ostomy	102	100.00%	0	0.00%
182904 Irrigation techniques (if applicable)	33	32.40%	69	67.60%
182905 How to measure stoma	100	98.00%	2	2.00%
182907 Complications related to stoma	101	99.00%	1	1.00%
182908 Schedule for changing ostomy bag	101	99.00%	1	1.00%
182909 Suplies required to are for ostomy	100	98.00%	2	2.00%
182911 Diet modifications	102	100.00%	0	0.00%
182912 Fluid intake requirements	102	100.00%	0	0.00%
182913 Odor control mechanisms	1	1.00%	101	99.00%
182914 Modifications of daily activities	100	98.00%	2	2.00%
182915 Procedure to change ostomy bag	100	98.00%	2	2.00%
2001 Spiritual health				
200122 Spiritual contentment	8	7.80%	94	92.20%
2008 Comfort Status				
200803 Psychological well-being	0	0.00%	102	100.00%
200806 Social support from family	0	0.00%	102	100.00%
2600 Family coping				
260007 Expresses feelings and emotions openly among members	2	2.00%	100	98.00%
260024 Uses available family support system	2	2.00%	100	98.00%
2602 Family functioning				
260210 Obtains adequate resources to meet needs of members	17	16.70%	85	83.30%
260213 Involves members in problem solving	17	16.70%	85	83.30%
2603 Family integrity				
260304 Members assist one another in performing roles and daily tasks	0	0.00%	102	100.00%
260306 Members share thoughts, feelings, interests, concerns	0	0.00%	102	100.00%

Thus, the following were present in all cases: the NOC “Participation in making Health Care Decisions (1606)” and its indicators “Seeks reputable information (160603)”, “Defines available options (160604)”, and “Identifies available support for achieving desired outcomes (160610)”, as well as the NOC “Knowledge: Ostomy Care (1829)” and its indicators “Skin care needs around ostomy (182903)”, “Diet modifications (182911)”, and “Fluid intake requirements (182912)”.

We analysed the prevalence of all factors, considering that the NOC outcome was present. The variables Sex, Being a member of an ostomy association, Time with stoma, and especially Period of care were significantly associated with a number of NOC outcomes and their indicators. The variables Family member with an ostomy, Medical diagnosis, and Stoma site marking did not show any significant association (Supplementary Material S1).

Table 3 shows the OR and CI (95%) values for the statistically significant associations between variables and indicators.

**Table 3:** OR of significant NOC outcomes and their indicators vs. factors.

NOC indicators	OR	CI (95%)
Sex (Female vs. Male)		
000404, 000421	2.500	1.027–6.089
011901	0.307	0.109–0.865
011907, 011908	0.366	0.135–0.988
011911	0.301	0.099–0.913
011912	0.333	0.109–1.018
011913, 011921	0.336	0.118–0.954
101503, 101524	8.302	0.982–70.158
120005, 120014	2.400	1.084–5.315
161519	5.488	1.123–26.826
161523	6.250	1.294–30.165
Ostomy association (No vs. Yes)		
011901	0.087	0.016–0.488
011907, 011908	0.093	0.016–0.521
011909	0.163	0.033–0.798
011910	0.026	0.002–0.345
011911	0.140	0.028–0.693
011912	0.129	0.026–0.642
011913, 011921	0.081	0.014–0.454
150303, 150311	0.163	0.033–0.799
Time (>1 year vs. <1 year)		
000404, 000421	0.245	0.071–0.849
011901	7.000	1.957–25.039
011907, 011908	6.475	1.821–23.022
011909	5.000	1.418–17.628
011911	3.877	1.076–13.966
011912	4.230	1.165–15.360
011913, 011921	5.000	1.418–17.628
182904	3.446	1.002–11.847
Period of care (Follow-up care vs. Postoperative care)		
100403, 100408, 100411	0.280	0.103– 0.765
110113, 110115, 110121	6.432	2.606–15.870
120005, 120014	2.265	1.018– 5.039
130906, 130915	0.150	0.060–0.378
140202, 140207, 140211, 140214	0.113	0.035–0.368
140210	0.054	0.011–0.252
150303, 150311	23.784	3.045–185.750
161519	9.361	1.150–76.172
161523	10.521	1.303–84.936
170102, 170107, 170108	0.097	0.011–0.820

*OR, Odds ratio; CI, Confidence Interval.*

### Descriptive Analysis of NOC Indicator Scores

The results obtained from the descriptive analysis of the NOC outcomes with 20 or more cases can be seen in Table 4. The scores for each indicator were compared with the rest of the factors. This analysis showed that Time with stoma and Period of care were the most influential variables in the study (Supplementary Material S2). Notably, Medical diagnosis did not show any significant associations, as in the previous bivariate analysis.

**Table 4:** Descriptive analysis of NOC outcomes with 20 or more cases.

NOC	M	SD	Me	IQR	CI (95%)
0004 Sleep					
000404 Sleep quality	2.75	0.550	3.00	2.00–3.00	2.62–2.88
000421 Difficulty getting to sleep	2.92	0.550	3.00	2.00–3.00	2.79–3.05
0006 Psychomotor Energy					
000608 Exhibits stable energy level	2.90	0.489	3.00	3.00–3.00	2.71–3.08
000609 Exhibits ability to accomplish daily tasks	3.28	0.649	3.00	3.00–4.00	3.03–3.52
0119 Sexual functioning					
011901 Attains sexual arousal	2.77	0.752	3.00	2.00–300	2.44–3.11
011907 Expresses ability to perform sexually despite physical imperfection	3.00	0.853	3.00	3.00–4.00	2.63–3.37
011908 Expresses comfort with sexual expression	2.70	0.703	3.00	2.00–3.00	2.39–3.00
011909 Expresses self-esteem	2.86	0.478	3.00	3.00–3.00	2.64–3.07
011913 Express willingness to be sexual	3.00	0.548	3.00	3.00–3.00	2.75–3.25
011921 Communicates comfortably with partner	3.05	0.509	3.00	3.00–3.00	2.78–3.33
1004 Nutritional Status					
100403 Energy	2.68	0.477	3.00	2.00–3.00	2.47–2.89
100408 Fluid intake	2.86	0.560	3.00	2.75–3.00	2.62–3.11
100411 Hydration	2.95	0.575	3.00	3.00–3.00	2.70–3.21
1101 Tissue integrity: Skin and Mucous Membranes					
110113 Skin integrity	3.15	0.504	3.00	3.00–3.00	3.03–3.28
110115 Skin Lesions	3.24	0.681	3.00	3.00–4.00	3.08–3.41
110121 Erythema	3.17	0.571	3.00	3.00–3.25	3.03–3.31
1200 Body Image					
120005 Satisfaction with body appearance	2.80	0.452	3.00	3.00–3.00	2.67–2.93
120014 Adjustment to body changes due to injury	3.04	0.450	3.00	3.00–3.00	2.91–3.17
1205 Self-Esteem					
120501 Verbalizations of self-acceptance	2.64	0.549	3.00	2.00–3.00	2.44–2.83
120502 Acceptance of self-limitations	2.76	0.502	3.00	2.00–3.00	2.58–2.94
1302 Coping					
130210 Uses behaviors to reduce stress	2.42	0.564	2.00	2.00–3.00	2.21–2.63
130212 Use effective coping strategies	2.57	0.573	3.00	2.00–3.00	2.35–2.79
130213 Avoids unduly stressful situations	2.68	0.541	3.00	2.00–3.00	2.48–2.88
1309 Personal Resiliency					
130906 Exhibits positive mood	2.50	0.564	3.00	2.00–3.00	2.30–2.70
130915 Proposes practical. constructive solutions for disputes	2.76	0.554	3.00	2.00–3.00	2.57–2.96
1402 Anxiety Self-Control					
140202 Eliminates precursor of anxiety	2.41	0.590	2.00	2.00–3.00	2.15–2.67
140207 Uses relaxation techniques to reduce anxiety	2.68	0.568	3.00	2.00–3.00	2.43–2.93
140210 Maintains role performance	2.50	0.688	2.5	2.00–3.00	2.18–2.82
140211 Maintains social relationships	2.68	0.568	3.00	2.00–3.00	2.43–2.93
140214 Maintains adequate sleep	2.73	0.550	3.00	2.00–3.00	2.48–2.97
1503 Social Involvement					
150303 Interacts with family members	2.95	0.218	3.00	3.00–3.00	2.85–3.05
150311 Participates in leisure activities with others	2.90	0.625	3.00	2.50–3.00	2.62–3.19
1606 Participation in Health Care Decisions					
160603 Seeks reputable information	3.14	0.758	3.00	3.00–4.00	2.99–3.29
160604 Defines available options	2.95	0.750	3.00	2.00–3.25	2.80–3.10
160610 Identifies available support for achieve desired outcomes	3.20	0.718	3.00	3.00–4.00	3.06–3.34
1829 Knowledge: Ostomy Care					
182901 Function of ostomy	2.78	1.006	3.00	2.00–4.00	2.58–2.98
182903 Skin care needs around ostomy	2.63	0.933	3.00	2.00–3.00	2.44–2.81
182904 Irrigation techniques (if applicable)	2.03	0.847	2.00	1.00–3.00	1.73–2.33
182905 How to measure stoma	2.68	0.942	3.00	2.00–3.00	2.49–2.87
182907 Complications related to stoma	2.54	0.843	3.00	2.00–3.00	2.38–2.71
182908 Schedule for changing ostomy bag	2.78	0.912	3.00	2.00–3.00	2.60–2.96
182909 Supplies required to are for ostomy	2.76	0.955	3.00	2.00–3.00	2.57–2.95
182911 Diet modifications	2.57	0.850	3.00	2.00–3.00	2.40–2.74
182912 Fluid intake requirements	2.61	0.834	3.00	2.00–3.00	2.44–2.77
182914 Modifications of daily activities	2.50	0.759	3.00	2.00–3.00	2.35–2.65
182915 Procedure to change ostomy bag	2.96	0.974	3.00	2.25–4.00	2.77–3.15

*M, Mean; SD, Standard Deviation; Me, Median; IQR, Interquartile Range; CI, Confidence Interval.*

## Discussion

The aim of this study was to determine NOC outcomes in patients with intestinal stoma and to identify their associations with sociodemographic and clinical variables. This study is in line with previous studies using NANDA diagnoses (16) and NIC interventions (15) in the same sample, and is therefore consistent with what has already been observed. This article completes the publication of the results obtained from the patient sample by analysing the three key elements of the nursing process, i.e. diagnoses, interventions, and outcomes.

Period of care is the most influential factor. Patients’ willingness to address their problem must be highlighted. Their knowledge and care of the stoma are also relevant aspects that must be included in the care plan from the time of the surgery and throughout the continuity of care. This implies including indicators related to these two aspects, which were found to be applicable to the sample in 100% of the cases. Nutritional status and anxiety should be assessed immediately after surgery. The assessment of indicators on body image, social involvement, and tissue integrity are particularly important in patient care and should be assessed, especially in the period of continuity of care, once the acute phase of the process is over. The other outcomes and indicators analysed failed to show a clear association in one period or another, suggesting that their inclusion in the care plan should be considered depending on the individual circumstances of each patient.

Another objective was to assess the status of the sample analysed on the basis of the scores obtained on the NOC indicators. The mean scores of the analysed indicators ranged between 2 and 3 (maximum = 5 points), indicating a moderate level of deterioration in patient condition. It is important to note that the scoring is fairly homogeneous between the indicators for the biological, psychological, and social status of the patients. It is important to highlight that score of the indicators improve when comparing the period of continuity of care versus the postoperative period, more significantly indicators on making health decisions and knowledge of stoma care**.**

There are few studies in which NANDA, NOC, or NIC nursing taxonomies have been used in patients with gastrointestinal stoma. As a result, it is difficult to discuss this study in comparison with previous research. Instead, we provide proposals for care plans and discuss the results of a number of clinical case studies.

The presence of an intestinal stoma can negatively affect patients’ quality of life, influencing their physical, psychological, and social spheres. This is consistent with the results of our study, in which the impact of the indicators extends to the different spheres of health. Regarding the physical sphere, once the stoma is created, the integrity of the skin must be preserved to ensure the correct adherence of the ostomy pouch to the body and to avoid complications that can occur if good care is not taken, such as irritation, fracturing, and peristomal pain. It is true that various studies report different incidence rates of patients with peristomal pain, which will depend on the individual characteristics of each patient. All of these complications can be reduced and even avoided (17). In this sense, recent studies propose the use of standardized algorithms and apps for the prevention and treatment of peristomal skin complications (18, 19).

Continuity of care after discharge reduces the occurrence of complications and improves quality of life over time. Also, marking the stoma site according to the anatomical characteristics of each individual and the information provided prior to surgery can lead to a lower incidence of complications (20). In fact, one of the strongest associations observed in our results shows that difficulties in falling asleep are less frequent in patients with stoma site marking.

The formation of a stoma after surgery has a negative impact on patients with intestinal stoma, influencing their postoperative period and thus their level of self-esteem. For a better adaptation to the new changes in the coping phase, patients should accept their own diagnosis. Adequate care by nursing professionals will have a positive impact on the patient, who will be able to promote self-care after discharge and avoid complications related to eating, inactivity, hygiene, and social interaction (21, 22). On the one hand, our results propose a work system that facilitates the planning of the outcomes to be measured as well as the rest of the care plan, taking into consideration the different phases through which the patient passes. Therefore, it is relevant to differentiate between immediate needs after surgery and other needs that can be addressed in the medium term. On the other hand, during the period of continuity of care, which is characterised by the care provided by a stoma therapy nurse in the consultation room, significant improvements have been observed in indicators related to resilience, anxiety, participation in making health decisions, and knowledge of stoma care.

The scarce presence of indicators related to social and family support is noteworthy. The data from this study provide little evidence on this issue, although family support is crucial for coping with the presence of the intestinal stoma and the disease. Family ties prior to the new change process are essential to fostering greater unity or separation in the family network when faced with an unfamiliar phenomenon. Once healthcare professionals have assessed the relationship between family members and the patient, they may offer them recommendations and advice (23). Our results suggest the need for ongoing assessment of indicators related to family integrity, family functioning, and ability to cope with problems that may arise in the family environment. Professionals should pay particular attention to the assessment of social involvement, which may manifest itself more significantly in the period of continuity of care.

Caregivers are another major gateway to the process of change, as they also have to adapt and cope with change, being the main support for patients. As such, they may also see their quality of life declining physically, psychologically and socially. Caregivers receive help and training, but only during the initial stage or during hospitalisation, and it is only later that the psychological distress and anxiety of seeing their relatives suffer emotionally arises. They feel overwhelmed by the burden of care, their worries, and the difficulty of taking care of their own health issues, so nursing professionals must include them in the care plans from the beginning to the end of care (24).

### Limitations

This study is not without limitations. Firstly, as this is an observational, cross-sectional study, we cannot verify whether the associations found reflect the true causal relationship between the variables, so they should be regarded as causal hypotheses that need to be tested in follow-up studies involving a larger sample size. Secondly, this study is based on a list of NOC outcomes developed from a previous study. As a result, relevant outcomes may have been left out of the study. This is partly countered by the expert peer review of the 30 NOC outcomes presented. Furthermore, the inclusion of each patient’s outcomes was supervised by a second observer, although it was not an on-site assessment, an aspect that will be improved in future research. Thirdly, the previous study is based on a comprehensive review of the published scientific literature on the needs expressed by ostomy patients.

Finally, new related factors could be included in future studies. For instance, stoma permanence is a relevant factor that was not explored in this study. Another aspect highlighted by other studies is the financial status and level of education of patients (25, 26), which could also be included in future research.

### Implications for Practice

The analysis of the Nursing Outcomes Classification in such a specific context can serve as a guide for helping nurses to develop individual care plans focused on patients with intestinal stomas. It can help to assess the impact of the interventions implemented by nurses. The availability of standardised outcomes is essential for documentation purposes in electronic records, for use in clinical information systems, for the advancement of nursing knowledge, and for the education of professional nurses and students. This study is thus intended to fill the gap in the existing literature regarding studies using the NOC in a specific care setting with these specific patients. Consequently, the implementation of these plans will ensure the good quality of care and the continuity of care, favouring the management of the disease process and benefitting the patient’s situation (27), while supporting the advanced practice model.

## Conclusion

In the development of a care plan for individuals with an intestinal stoma, it is relevant to include indicators measuring their participation in making decisions related to their condition, as well as other indicators related to their knowledge and self-care of the stoma. It is also relevant to address their psychosocial needs, including their related indicators, while taking into consideration the individualisation of care necessary in the nursing process.

Based on the scores obtained from the NOC indicators analysed, it can be concluded that the physical, psychological, and social spheres of the individuals with an ostomy are clearly impaired. The data obtained show a moderate degree of impairment in the indicators analysed, which is also associated with the period of care that the patient is undergoing and, consequently, with when the stoma was performed.

## Data Availability

The raw data supporting the conclusions of this article will be made available by the authors, without undue reservation.
