# Supplementary material for: Assessment of Health Indicators in Individuals with Intestinal Stoma using the Nursing Outcomes Classification: A Cross-Sectional Study
Source: Front Surg. 2022 May 20;9:870379. doi: 10.3389/fsurg.2022.870379 (PMC9163492; doi:10.3389/fsurg.2022.870379)
Supplement: Supplementary file 1 [file Data_Sheet_1_v1.pdf]

frontiers

Supplementary Material

Assessment of health indicators in individuals with intestinal stoma using the Nursing Outcomes Classification. A cross-sectional study

Noelia Moya-Muñoz[1], Elena Armenteros-Fernández[2], Clara Bautista-Mártir[3], Irene Del Pilar Vílchez-Díaz[4], Isabel María López-Medina[5], Rafael Montoya-Juárez[6], César Hueso-Montoro[6*], Concepción Capilla-Díaz[6].

[1]Virgen de las Nieves University Hospital, Granada, Spain

[2]Son Espasses University Hospital, Palma de Mallorca, Spain

[3]Virgen de la Luz Hospital, Cuenca, Spain

[4]Toledo University Hospital, Toledo, Spain

[5]Department of Nursing, Faculty of Health Sciences, University of Jaen, Jaen, Spain

[6]Department of Nursing, Faculty of Health Sciences, University of Granada; Instituto de Investigación Biosanitaria ibs.GRANADA, Granada, Spain.

*** Correspondence:**
César Hueso-Montoro
cesarhueso@ugr.es

Supplementary Material 1. Association between NOC indicators and factors

		Age		Sex			Ostomy association			Ostomy in the family			Medical diagnosis			Time (years)			Stoma marking			Period of care		
		M±SD	p	f	f	p	f	f	p	f	f	p	f	f	p	f	f	p	f	f	p	f	f	p
**NOC indicators**				**M**	F		**Y**	**N**		**Y**	**N**		**O**	**NoO**		**<1**	**>1**		**Y**	**N**		**PO**	**FU**	
000404, 000421	Y	63.69±10.58	0.120	32	40	0.041*	4	68	0.417	12	60	0.773	57	15	0.629	67	5	0.038*	35	37	0.157	60	12	0.687
	N	59.87±12.65		20	10		3	27		4	26		25	5		23	7		10	20		24	6	
000608, 000609	Y	61.31±13.07	0.481	12	17	0.221	2	27	1.000	3	26	0.547	22	7	0.468	29	0	0.018*	24	5	0.946	16	13	0.156
	N	63.07±10.57		40	33		5	68		13	60		60	17		61	12		60	13		29	44	
011901	Y	63.91±9.26	0.533	16	6	0.021*	5	17	0.005*	4	18	0.744	19	3	0.553	15	7	0.003*	18	4	1.000	0	22	<0.001*
	N	62.20±11.82		36	44		2	78		12	68		63	17		75	5		66	14		45	35	
011907, 011908	Y	63,70±9,108	0.589	16	7	0.043*	5	18	0.006*	4	19	0.754	20	3	0.552	16	7	0.005*	19	4	1.000	0	23	<0.001*
	N	62.24±11.89		36	43		2	77		12	67		62	17		74	5		65	14		45	34	
011909	Y	64.14±9.38	0.476	14	7	0.107	4	17	0.031*	4	17	0.737	19	2	0.234	15	6	0.016*	18	3	0.759	0	18	<0.001*
	N	62.26±11.76		38	43		3	78		12	69		63	18		75	6		66	15		45	36	
011910	Y	64.33±5.03	0.785	1	2	0.614	2	1	0.012*	0	3	1.000	3	0	1.000	2	1	0.316	3	0	1.000	0	3	0.253
	N	62.52±11.44		51	48		5	94		16	83		79	20		88	11		81	18		45	54	
011911	Y	61.89±8.32	0.775	14	5	0.028*	4	15	0.022*	3	16	1.000	17	2	0.351	14	5	0.045*	17	2	0.514	0	45	<0.001*
	N	62.72±11.91		38	45		3	80		13	70		65	18		76	7		67	16		19	38	
011912	Y	62.20±8.44	0.887	13	5	0.047*	4	14	0.017*	3	15	1.000	16	2	0.514	13	5	0.035*	16	2	0.733	0	18	<0.001*
	N	62.64±11.86		39	45		3	81		13	71		66	18		77	7		68	16		45	39	
011913, 011921	Y	62.43±8.26	0.950	15	6	0.035*	5	16	0.004*	3	18	1.000	18	3	0.758	15	6	0.016*	18	3	0.759	0	21	<0.001*
	N	62.60±12.01		37	44		2	79		13	68		64	17		75	6		66	15		45	36	
050101	Y	65.33±8.02	0.669	0	3	0.114	1	2	0.194	0	3	1.000	3	0	1.000	3	0	1.000	3	0	1.000	1	2	1.000
	N	62.48±11.40		52	47		6	93		16	83		79	20		87	12		81	18		44	55	
100403, 100408, 100411	Y	59.73±14.50	0.184	8	14	0.121	1	21	1.000	2	20	1.000	17	5	0.763	21	1	0.454	20	2	0.518	15	7	0.010*
	N	63.35±10.22		44	36		6	74		14	66		65	15		69	11		64	16		30	50	
101501	Y	54.00±16.82	0.184	0	3	0.121	0	3	1.000	0	3	0.512	2	1	0.763	3	0	0.454	3	0	0.348	3	0	0.010*
	N	62.83±11.11		52	47		7	92		16	83		80	19		87	12		81	18		42	57	
101503, 101524	Y	59.00±12.78	0.355	1	7	0.030*	0	8	1.000	0	8	0.351	7	1	0.763	8	0	0.591	7	1	1.000	5	3	0.297
	N	62.87±11.19		51	43		7	87		16	78		75	19		82	12		77	17		40	54	
110113, 110115, 110121	Y	63.02±11.43	0.591	35	31	0.575	5	61	1.000	11	5	0.712	56	10	0.125	61	5	0.107	55	11	0.725	19	47	<0.001*
	N	61.75±11.18		17	19		2	34		5	31		26	10		29	7		29	7		26	10	
120005, 120014	Y	63.10±11.75	0.644	2	30	0.030*	4	30	0.713	6	46	0.315	38	12	0.273	45	5	0.588	43	7	0.343	17	33	0.004*
	N	62.06±10.94		32	20		3	49		10	42		44	8		45	7		41	11		28	24	
120501, 120502	Y	62.21±12.16	0.827	14	19	0.232	2	31	1.000	7	26	0.289	26	7	0.778	32	1	0.097	26	7	0.518	16	17	0.539
	N	62.74±10.95		38	31		5	64		9	60		56	13		58	11		58	11		29	40	
130002, 130010	Y	58.80±13.59	0.447	2	3	0.675	0	5	1.000	1	4	0.582	3	2	0.252	5	0	1.000	5	0	0.514	13	2	0.583
	N	62.76±11.22		50	47		7	90		15	82		79	18		85	12		79	18		42	55	
130210, 130213	Y	63,77±10,93	0.479	13	18	0,227	2	5	1.000	5	11	1.000	24	7	0.617	31	0	0.016*	26	5	0.790	15	16	0.566
	N	62,04±11,49		39	32		29	66		26	60		58	13		59	12		58	13		30	41	
130212	Y	63.61±11.30	0.571	13	39	0,572	1	27	0.670	5	23	0.763	21	7	0.399	28	0	0.033*	23	5	1.000	14	14	0.462
	N	62.04±11.49		15	35		6	68		11	63		61	13		62	12		61	13		31	43	
130411, 130414	Y	61.50±6.36	0.893	0	2	0.238	1	1	0.133	0	2	1.000	2	0	1.000	2	0	1.000	2	0	1.000	8	2	0.502
	N	62.59±11.39		52	48		6	94		16	84		80	20		88	12		82	18		45	55	
130906, 130915	Y	61.24±10.54	0.402	16	8	0.575	2	32	1.000	3	31	0.178	27	7	0.860	34	0	0.008*	29	5	0.582	25	9	<0.001*
	N	63.24±11.68		36	32		5	63		13	55		55	13		56	12		55	13		20	48	

140202, 140207, 140211, 140214	Y	63.23±8.74	0.759	10	12	0.558	1	21	1.000	4	18	0.744	17	5	0.763	22	0	0.065	21	1	0.111	18	4	<0.001*
	N	62.39±11.95		42	38		6	74		12	68		65	15		67	12		63	17		27	53	
140210	Y	63.40±9.05	0.716	10	10	0.922	0	20	0.340	4	12	0.512	15	5	0.535	20	0	0.117	19	1	0.518	18	2	<0.001*
	N	62.37±11.82		42	40		7	75		14	70		67	15		70	12		65	17		27	55	
140401, 140402, 140406, 140407	Y	59.00±8.76	0.522	3	1	0.618	4	1	1.000	1	3	0.500	2	2	0.172	4	0	1.000	4	4	1.000	3	1	0.318
	N	62.71±11.40		49	49		91	15		15	83		80	18		86	12		80	18		42	56	
140405	Y	57.00±9.54	0.389	2	1	1.000	0	3	1.000	1	2	0.404	2	1	0.484	3	0	1.000	3	0	1.000	3	0	0.083
	N	62.74±11.35		50	49		7	92		15	84		80	19		87	12		81	18		42	57	
140410, 140411	Y	65.00	0.830	1	0	1.000	0	1	1.000	0	1	1.000	0	1	0.196	1	0	1.000	1	0	1.000	0	1	0.318
	N	62.54±11.35		51	50		7	94		16	85		82	19		89	12		83	18		45	56	
150303, 150311	Y	66.38±10.72	0.083	10	11	0.730	4	17	0.031*	4	17	0.737	18	3	0.758	16	5	0.120	18	3	0.759	1	20	<0.001*
	N	61.58±11.30		42	39		3	78		12	69		64	17		74	7		66	15		44	37	
161519	Y	61.00±10.02	0.628	2	9	0.021*	2	9	0.165	1	10	1.000	9	2	1.000	11	0	0.353	9	2	1.000	1	10	0.021*
	N	62.76±11.48		50	41		5	86		15	76		73	18		79	12		75	16		44	47	
161523	Y	62.92±11.63	0.910	2	10	0.011*	2	10	0.191	1	11	0.685	10	72	1.000	11	1	1.000	9	3	0.440	1	11	0.008*
	N	62.52±11.32		50	40		5	85		15	75		72	18		79	11		75	15		44	46	
170102, 170107, 170108	Y	62.50±11.12	0.986	5	3	0.716	0	8	1.000	2	6	0.608	6	2	0.653	8	0	0.591	7	1	1.000	7	1	0.020*
	N	62.57±11.38		47	47		7	87		14	80		76	18		82	12		77	17		38	17	
170104	Y	65.75±6.99	0.568	3	1	0.618	0	4	1.000	0	4	1.000	2	2	0.172	4	0	1.000	3	3	0.546	3	1	0.318
	N	62.44±11.45		49	49		7	91		16	82		80	18		86	12		81	17		42	56	
182901	Y	62.63±11.33	0.562	51	50	1.000	7	94	1.000	16	85	1.000	81	20	1.000	89	12	1.000	83	18	1.000	45	56	1.000
	N	56.00		1	0		0	1		0	1		1	0		1	0		1	0		0	1	
182904	Y	59.36±12.22	0.061	21	12	0.077	4	29	0.209	5	28	0.918	25	8	0.415	26	7	0.053*	26	7	0.154	16	17	0.539
	N	64.10±10.58		31	38		3	66		11	58		57	12		64	5		58	11		29	40	
182905, 182909	Y	62.42±11.18	0.350	51	49	1.000	7	93	1.000	16	84	1.000	80	20	1.000	89	1	0.222	83	17	0.323	45	55	0.502
	N	70.00±19.79		1	1		0	2		0	2		2	0		1	1		1	1		0	2	
182907	Y	62.53±11.35	0.762	51	50	1.000	7	94	1.000	16	85	1.000	82	19	0.196	89	12	1.000	83	18	1.000	44	57	0.441
	N	66.00		1	0		0	1		0	1		0	1		1	0		1	0		1	0	
182908	Y	62.63±11.33	0.562	51	50	1.000	7	94	1.000	16	85	1.000	81	20	1.000	89	12	1.000	83	18	1.000	45	56	1.000
	N	56.00		1	0		0	1		0	1		1	0		1	0		1	0		1	1	
182913	Y	48.00	0.197	1	0	1.000	0	1	1.000	0	1	1.000	0	1	0.196	1	0	1.000	1	0	0.323	1	0	0.441
	N	62.71±11.26		51	50		7	94		16	85		82	19		89	12		83	18		44	57	
182914, 182915	Y	62.61±11.39	0.795	50	50	0.495	7	93	1.000	16	84	1.000	81	19	0.355	88	12	1.000	82	18	1.000	45	55	0.502
	N	60.50±6.36		2	0		0	2		0	2		1	1		2	0		2	0		0	2	
200122	Y	65.75±10.33	0.410	3	5	0.483	1	7	0.446	2	6	0.608	7	1	1.000	6	2	0.238	7	1	1.000	3	5	1.000
	N	62.30±11.39		49	45		6	88		14	80		75	19		84	10		77	17		42	52	
260007, 260024	Y	61.50±6.36	0.893	0	2	0.238	1	1	0.133	0	2	1.000	2	0	1.000	2	0	1.000	2	0	1.000	0	2	0.502
	N	62.59±11.39		52	48		6	94		16	84		80	20		88	12		82	18		45	55	
260210, 260213	Y	63.24±9.86	0.791	10	7	0.479	0	17	0.597	2	15	1.000	12	5	0.316	17	0	0.209	13	4	0.494	8	9	0.789
	N	62.44±11.61		42	43		7	78		14	71		70	15		73	12		72	14		37	48	

M=Mean; SD=Standard Deviation; M=Male; F=Female; Y=Yes; N=No; O=Oncological; NoO=Non-oncological; PO=Postoperative care; FU=Follow-up care; Y=Yes; N=No. * Significant *p*-value

Supplementary Material 2. Association between the scores for significant NOC indicators with 20 or more cases and their factors

Indicator	Category	N	M	SD	Me	IQR	p
**Ostomy in the family**							
011921	Yes	3	3.67	0.577	4.00	3.00-4.00	0.047
	No	18	2.94	0.539	3.00	3.00-3.00	
120005	Yes	6	3.17	0.408	3.00	2.74-3.60	0.046
	No	44	2.75	0.438	3.00	2.62-2.88	
140214	Yes	4	2.25	0.500	2.00	1.45-3.05	0.049
	No	18	2.83	0.514	3.00	2.58-3.09	
**Ostomy association**							
000421	Yes	4	2.25	0.500	2.00	2.00-2.75	0.011
	No	68	2.96	0.531	3.00	3.00-3.00	
150303	Yes	4	2.75	0.500	3.00	1.95-3.55	0.039
	No	17	3.00	ct	3.00	3.00-3.00	
**Time (years)**							
011901	<1	15	3.00	0.655	3.00	3.00-3.00	0.047
	>1	7	2.29	0.756	2.00	2.00-3.00	
182901	<1	89	2.67	1.009	3.00	2.00-4.00	0.003
	>1	12	3.58	0.515	4.00	3.00-4.00	
182903	<1	90	2.54	0.950	3.00	2.00-3.00	0.010
	>1	12	3.25	0.452	3.00	2.00-3.75	
182904	<1	26	1.85	0.834	2.00	1.00-3.00	0.016
	>1	7	2.71	0.488	3.00	2.00-3.00	
182905	<1	89	2.56	0.916	3.00	2.00-3.00	<0.001
	>1	11	3.64	0.505	4.00	3.00-4.00	
182907	<1	89	2.44	0.825	3.00	2.00-3.00	<0.001
	>1	12	3.33	0.492	3.00	3.00-4.00	
182908	<1	89	2.69	0.912	3.00	2.00-3.00	0.020
	>1	12	3.50	0.522	3.50	3.00-4.00	
182909	<1	89	2.65	0.943	3.00	2.00-3.00	0.001
	>1	11	3.64	0.505	4.00	3.00-4.00	
182911	<1	90	2.49	0.864	3.00	2.00-3.00	0.006
	>1	12	3.17	0.389	3.00	3.00-3.00	
182912	<1	90	2.52	0.838	3.00	2.00-3.00	0.003
	>1	12	3.25	0.452	3.00	3.00-3.75	
182914	<1	86	2.40	0.736	3.00	2.00-3.00	<0.001
	>1	12	3.25	0.452	3.00	3.00-3.75	
182915	<1	88	2.88	0.992	3.00	2.00-4.00	0.016
	>1	12	3.58	0.515	4.00	3.00-4.00	
**Period of care**							
130906	Postoperative care	25	2.36	0.569	2.00	2.13-2.59	0.013
	Follow-up care	9	2.89	0.333	3.00	2.63-3.15	
130915	Postoperative care	25	2.64	0.569	3.00	2.41-2.87	0.026
	Follow-up care	9	3.11	0.333	3.00	2.85-3.37	
140211	Postoperative care	18	2.56	0.511	3.00	2.30-2.81	0.039
	Follow-up care	4	3.25	0.500	3.00	2.45-4.05	
160603	Postoperative care	45	2.69	0.596	3.00	2.51-2.87	<0.001
	Follow-up care	57	3.49	0.685	4.00	3.31-3.67	
160604	Postoperative care	45	2.56	0.624	3.00	2.37-2.74	<0.001
	Follow-up care	57	3.26	0.695	3.00	3.08-3.45	
160610	Postoperative care	45	2.71	0.589	3.00	2.53-2.89	<0.001
	Follow-up care	57	3.58	0.565	4.00	3.43-3.73	
182901	Postoperative care	45	1.98	0.783	2.00	1.00-2.00	<0.001
	Follow-up care	46	3.43	0.628	3.50	3.00-4.00	
182903	Postoperative care	45	1.96	0.796	2.00	1.00-2.00	<0.001
	Follow-up care	57	3.16	0.649	3.00	3.00-4.00	
182904	Postoperative care	16	1.38	0.619	1.00	1.00-2.00	<0.001
	Follow-up care	17	2.65	0.493	3.00	2.00-3.00	
182905	Postoperative care	45	1.98	0.812	2.00	1.00-2.00	<0.001
	Follow-up care	55	3.25	0.584	3.00	3.00-4.00	
182907	Postoperative care	44	1.95	0.806	2.00	1.00-3.00	<0.001
	Follow-up care	57	3.00	0.535	3.00	3.00-3.00	
182908	Postoperative care	45	2.16	0.903	2.00	1.00-3.00	<0.001
	Follow-up care	56	3.29	0.530	3.00	3.00-4.00	
182909	Postoperative care	45	2.07	0.863	2.00	1.00-3.00	<0.001
	Follow-up care	55	3.33	0.579	3.00	3.00-4.00	

182911	Postoperative care	45	1.93	0.720	2.00	1.00-2.00	<0.001
	Follow-up care	57	3.07	0.563	3.00	3.00-3.00	
182912	Postoperative care	45	2.00	0.739	2.00	1.00-3.00	<0.001
	Follow-up care	57	3.09	0.544	3.00	3.00-3.00	
182914	Postoperative care	45	1.91	0.668	2.00	1.00-2.00	<0.001
	Follow-up care	55	2.98	0.408	3.00	3.00-3.00	
182915	Postoperative care	45	2.24	0.908	2.00	1.00-3.00	<0.001
	Follow-up care	55	3.55	0.538	4.00	3.00-4.00	
**Stoma site marking**							
000421	Yes	60	2.98	0.537	3.00	3.00-3.00	0.021
	No	12	2.58	0.515	3.00	2.00-3.00	
182907	Yes	83	2.46	0.845	3.00	2.00-3.00	0.045
	No	18	2.94	0.725	3.00	2.00-3.25	

M: Mean; SD: Standard Deviation; Me: Median; IQR: Interquartile Range; ct: constant
